# Association of lncRNA SH3PXD2A-AS1 with preeclampsia and its function in invasion and migration of placental trophoblast cells

**DOI:** 10.1038/s41419-020-02796-0

**Published:** 2020-07-27

**Authors:** Qian Chen, Sijia Jiang, Haihua Liu, Yue Gao, Xiaoxue Yang, Zhonglu Ren, Yunfei Gao, Lu Xiao, Haoyue Hu, Yanhong Yu, Xinping Yang, Mei Zhong

**Affiliations:** 1 Department of Obstetrics and Gynecology, Nanfang Hospital, Southern Medical University, Guangzhou, 510515 China; 2 Key Laboratory of Mental Health of the Ministry of Education, Southern Medical University, Guangzhou, 510515 China; 3 Department of Bioinformatics, School of Basic Medical Sciences, Southern Medical University, Guangzhou, 510515 China

**Keywords:** Disease model, Non-coding RNAs

## Abstract

Accumulating evidence suggests that the pathogenesis of preeclampsia involves poor placentation caused by insufficient trophoblast invasion and impaired uterine spiral artery remodeling, yet the underlying molecular mechanism remains unclear. We carried out transcriptome profiling on placentae from preeclamptic patients and normal subjects, and identified about four hundred long non-coding RNAs differentially expressed in placentae of patients with early-onset severe preeclampsia. Here, we report our identification of lncRNA SH3PXD2A-AS1 as a potential causal factor for this disease and its downstream pathways involved in placentation. We found that expression level of SH3PXD2A-AS1 in the placentae is positively correlated with clinical severity of the patients. We demonstrated that SH3PXD2A-AS1 inhibited invasion and migration through recruiting CCCTC-binding factor (CTCF) to the promoters of SH3PXD2A and CCR7 to inhibit their transcription. Therefore, we conclude that the upregulation of lncRNA SH3PXD2A-AS1 may contribute to the pathogenesis of preeclampsia through prohibiting trophoblast invasion during placentation.

## Introduction

Preeclampsia (PE) is a severe pregnancy complication, characterized by new-onset hypertension and proteinuria after 20 weeks of gestation, often accompanied by signs of CNS symptoms, renal injury, or liver dysfunction^1^. The incidence rate is 4–5% of pregnancies worldwide^2^, which accounts for half of maternal perinatal morbidity^3^. PE can give rise to multiple organ injury due to associated vasoconstriction, microangiopathy, and malperfusion, especially eclampsia which result in tonic-clonic seizure^4^. PE also has significant consequences on fetal development and growth^5^, contributing to fetal growth restriction, placental abruption, and oligohydramnios^6^. Because the underlying mechanism remains unclear, there is no effective early diagnosis, nor radical treatment for PE^7–10^.

The suggested multifarious pathogenesis for PE includes deficient decidualization, immune response dysregulation, shallow trophoblast invasion, impaired spiral artery remodeling, and poor placental oxygen supply^5,11,12^. Numerous studies have suggested that dysregulated decidual cell function during the pathogenesis of preeclampsia may compromise trophoblast invasion and spiral artery remodeling prior to the traditional two-stage model^13–15^. The widely accepted two-stage model includes:^16^ (1) in early gestation, the poor trophoblast invasion and incomplete vascular abnormal formation of spiral arteries result in placenta dysfunctions; and (2) the dysfunctional placentae release factors into the maternal blood, which, in turn, cause hypertension and organ damage. Accumulating evidence supports this two-stage disease model, and it has been improved to have more details and finer classification of stages^17^. Therefore, the impairment of uterine spiral artery remodeling caused by insufficient trophoblast cells infiltration might be critical in early stage of PE development^18^. Shallow invasion of the trophoblasts into the endometrium and the placental vascular recasting barriers cause insufficient oxygen supply, leading to placental tissue ischemia hypoxia and endothelial cell damage^19^. Nevertheless, the exact molecular mechanism of PE remains unclear.

There are some similarities between placenta implantation and the growth of cancer cells. Both trophoblast and cancer cells can invade, migrate, and repress the immune reaction^20–22^. Numerous studies have revealed key roles of some long non-coding RNAs (lncRNAs) in the invasion and migration of cancer cells^23–25^. Some lncRNAs, such as H19^26^, HOTIAR^27^, MALAT1^28^, SPRY4-IT1^29^, and MEG3^30^, are reported to regulate proliferation, invasion, migration and apoptosis of placental trophoblasts. These processes are crucial for placenta development. A recent study shows that the lncRNA00473 is involved in the pathogenesis of PE through recruiting LSD1 to inhibit the expression of TFPI2, which in turn promotes the proliferation, migration, invasion and cellular network formation in trophoblast cells^31^. Another study demonstrates that the lncRNA HOXA11-AS promotes cell invasion and proliferation of trophoblast cells through recruiting Ezh2 and Lsd1 to regulate the expression of RND3 in the nucleus, and binding miR-15b-5p in the cytoplasm to modulate *HOXA7* mRNA expression^32^. These studies suggest that lncRNAs may play a crucial role in the pathogenesis of PE. Therefore, a systematic search for the placental lncRNAs associated with PE is needed in order to illustrate the molecular mechanism.

We have recently carried out a transcriptome sequencing on placentae from 9 patients with early-onset severe preeclampsia (EOSPE) and 32 normal controls and identifies 383 lnRNAs differentially expressed. We found that SH3PXD2A-AS1 was among the top differentially expressed lncRNAs. Here, we report our identification of lncRNA SH3PXD2A-AS1 as a potential causal factor for PE and its downstream pathways involved in placentation. We firstly verified its upregulation in the placentae of PE patients, and found that the upregulated expression of SH3PXD2A-AS1 is positively correlated with clinical severity. We further demonstrated that SH3PXD2A-AS1 inhibited invasion and migration through recruiting CTCF to the promoters of SH3PXD2A and CCR7 to inhibit their transcription. Therefore, we conclude that the upregulation of lncRNA SH3PXD2A-AS1 may lead to PE through prohibiting trophoblast invasion and migration during placentation.

## Results

### The lncRNA SH3PXD2A-AS1 is upregulated in preeclamptic placentae

**Fig. 1:**
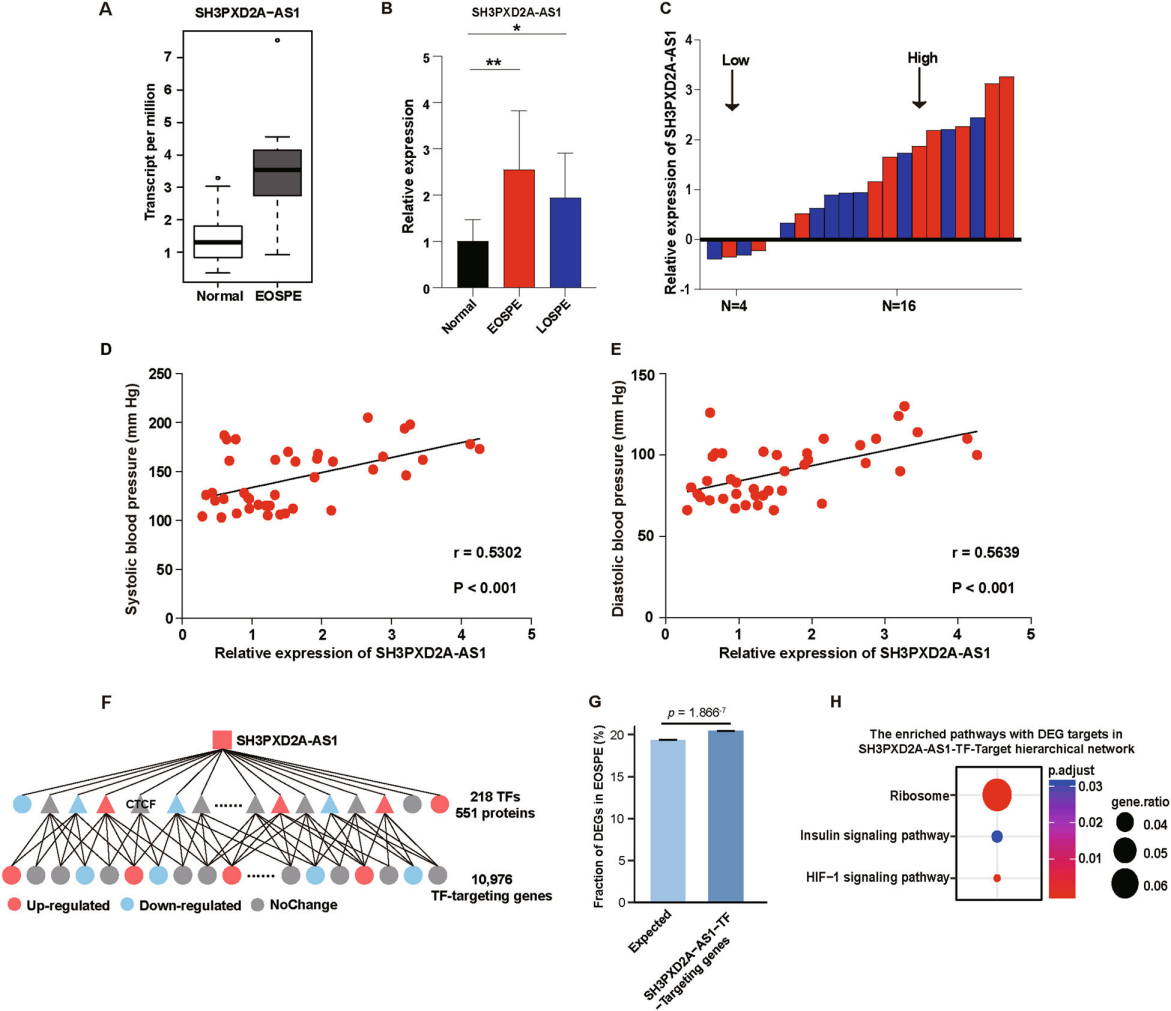
SH3PXD2A-AS1 as a potential PE-causal factor. **a** Boxplot of SH3PXD2A-AS1 expression levels in EOSPE placentae (*n* = 9) compared with normal placentae (*n* = 32). TPM: transcript per million. **b** The relative expression level of SH3PXD2A-AS1 measured by qRT-PCR. *Y*-axis: the fold-change; black bar: normal placentae, *n* = 20; red bar: placentae of patients with EOSPE, *n* = 10; blue bar: placentae of patients with LOSPE, *n* = 10; The values are shown as the mean ± S.D; **p < 0.01, *p < 0.05. **c** The expression level of SH3PXD2A-AS1 is upregulated in 80% (8/10) of EOSPE or LOSPE samples compared with the average level of 20 normal samples. Average expression level in normal samples was set as zero; the red bars represent expression levels in placentae of EOSPE patients; the blue bars represent expression levels in placentae of LOSPE patients. **d** Positive correlation (*r* = 0.5302, *p* < 0.001) between systolic blood pressure and the relative expression of SH3PXD2A-AS1 (Pearson correlation analysis). **e** Positive correlation (*r* = 0.5639, *p* < 0.001) between diastolic blood pressure and the relative expression of SH3PXD2A-AS1 (Pearson correlation analysis). **f** Hierarchical “SH3PXD2A-AS1-TF-Target” interaction network. **g** The targets in “SH3PXD2A-AS1-TF-Target” interaction network are enriched with DEGs in EOSPE. **h** The enriched pathways with DEGs as targets of “SH3PXD2A-AS1-TF-Target” interaction network.

We carried out transcriptome profiling on 41 placental samples, 9 from EOSPE and 32 from normal controls^33^, and found 383 differentially expressed lncRNAs. Most of the differentially expressed lncRNAs (DElncRNA) were antisense and long intergenic non-coding RNAs (lincRNAs) biotypes (Table S1). LncRNA SH3PXD2A-AS1 was among the top DElncRNAs that were significantly upregulated in the placenta of EOSPE patients (Fig. 1a). In order to verify the increased expression of SH3PXD2A-AS1 in patients, we collected placenta samples from 20 PE patients (10 early-onset severe PE (EOSPE) and 10 late-onset severe PE (LOSPE)) and 20 normal subjects. These patients showed significant difference in blood pressure and proteinuria (Table 1, Table S2). We performed qRT-PCR to detect the expression level of SH3PXD2A-AS1 in newly collected placentae from patients, and verified that both EOSPE patients and LOSPE patients showed significant increase in the expression of SH3PXD2A-AS1 (Fig. 1b). SH3PXD2A-AS1 was upregulated in 80% (8/10) of EOSPE or LOSPE samples compared to the average expression level of 20 normal samples (Fig. 1c). The expression level of SH3PXD2A-AS1 was positively correlated with the systolic and diastolic blood pressure (Fig. 1d, e). We searched for empirical DElncRNA–protein interactions from starBase, RAID, and NPInter, and also predicted DElncRNA–protein interactions using *cat*RAPID omics, and we obtained 551 SH3PXD2A-AS1–protein interactions, including 218 transcription factors (TFs) (Fig. 1f, Table S3). These TFs target 10,976 genes, which are significantly enriched with differentially expressed genes in EOSPE (Fig. 1g, Fisher’s exact test, *p* = 1.866^–7^), suggesting that SH3PXD2A-AS1 may be involved in the gene dysregulation in EOSPE. We further performed pathway enrichment analysis on the differentially expressed targets, and found 3 significantly enriched pathways, including ribosome, HIF-1 signaling pathway and insulin signaling pathway (Fig. 1h). HIF-1 signaling pathway is reported to be involved in invasion and migration in cancer^34^. Insulin signaling pathway is also found to be involved in invasion and migration in cancer^35,36^ and is reported to be associated with PE^37,38^. Some ribosomal proteins are found to be differentially expressed in cultured trophoblast cells treated with low or high concentration of oxygen^39^.

**Table 1:** Clinical characteristics of preeclamptic and normal pregnancies.

Variable	PE (*n* = 20)	Control (*n* = 20)	*p* value
Maternal age (year)	31.30 ± 5.741	31.15 ± 3.870	0.923
Maternal weight (kg)	70.28 ± 7.020	65.73 ± 7.399	0.053
Systolic blood pressure (mm Hg)	170.70 ± 16.762	115.35 ± 8.530	9.903E−16
Diastolic blood pressure (mm Hg)	105.00 ± 11.383	74.75 ± 5.812	6.876E−13
Proteinuria (g/day)	6.12 ± 4.820	--	--
Body weight of infant (g)	2299.5 ± 870.822	3148.0 ± 285.336	1.855E−4
Gestational age (weeks)	35.84 ± 3.291	39.31 ± 0.925	5.531E−5
Placental weight (g)	478.00 ± 83.009	525.00 ± 31.035	0.023
Fetal sex (Male)	11	9	0.527
Fetal sex (Female)	9	11	0.527
Primipara (cases)	9	8	0.749
Pluripara (cases)	11	12	0.749

### SH3PXD2A-AS1 inhibits invasion, migration and proliferation, and promotes cell death in trophoblast cells

**Fig. 2:**
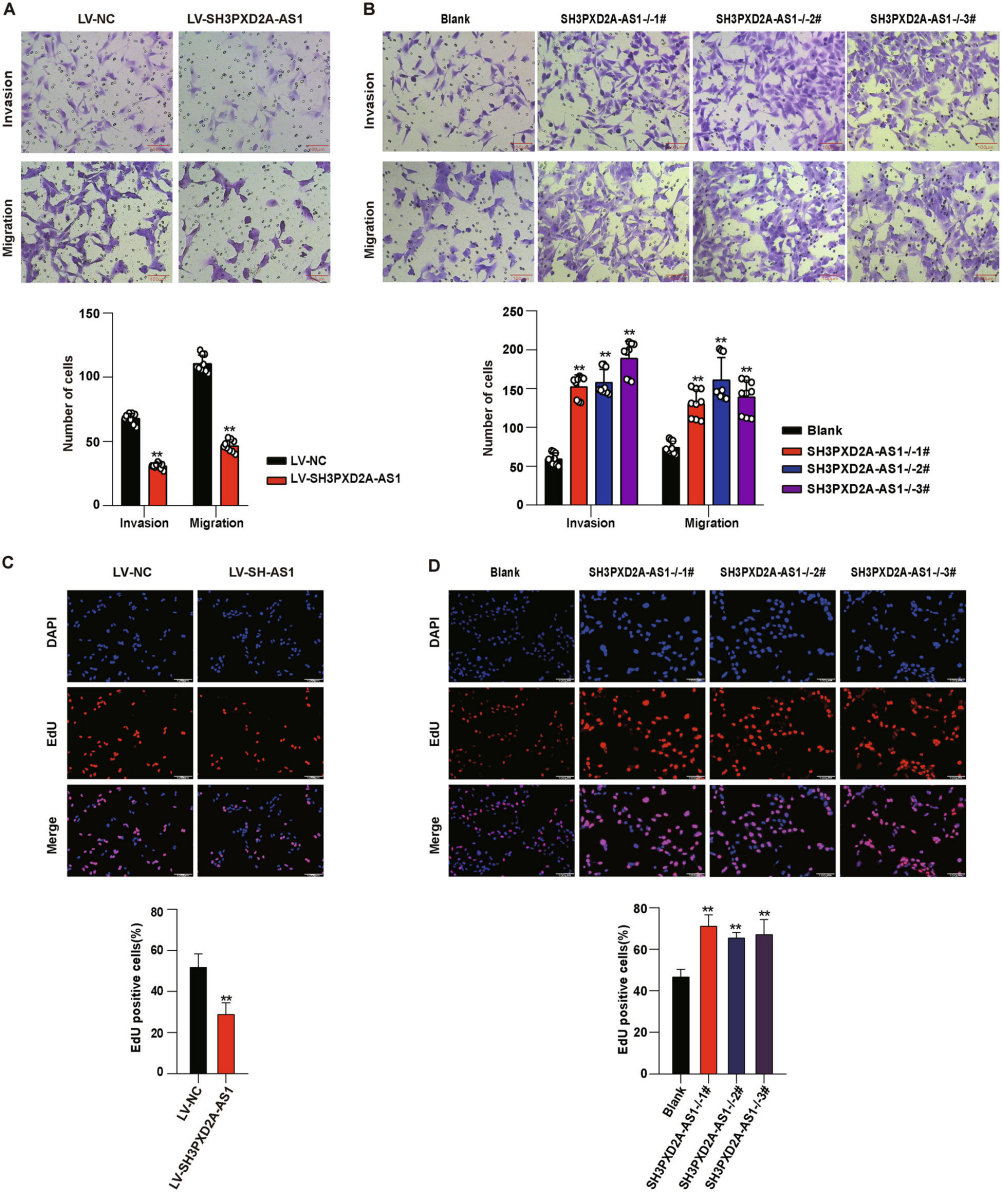
The effect of SH3PXD2A-AS1 on invasion, migration, and proliferation in HTR8/SVneo cells. **a** Both decreased migration and invasion were detected by Transwell assay in HTR8/SVneo cells after SH3PXD2A-AS1 overexpression. Upper panel: photos of cells stained with crystal violet; lower panel: statistics of cells. **b** Both increased migration and invasion were detected by Transwell assay in HTR8/SVneo cells after SH3PXD2A-AS1 knockout. Upper panel: pictures of cells stained with crystal violet; lower panel: statistics of cells. **c** Decreased proliferation was detected in HTR8/SVneo cells after SH3PXD2A-AS1 overexpression using EdU staining. Upper panel: pictures of cells stained with DAPI and EdU; lower panel: statistics of EdU positive cells. **d** Increased proliferation was detected by EdU staining in HTR8/SVneo cells after SH3PXD2A-AS1 knockout. Upper panel: pictures of cells stained with DAPI and EdU; lower panel: statistics of EdU positive cells. **p < 0.01, *p < 0.05.

The invasion and migration are critical in trophoblast infiltration and uterine spiral artery remodeling during placentation. In order to investigate the effect of SH3PXD2A-AS1 on invasion, migration, and proliferation of trophoblast cells, we established overexpression of SH3PXD2A-AS1 (Fig. S1A), and performed knockout of this lncRNA using CRISPR/Cas9 (Fig. S1B) in HTR8/SVneo cells. We performed Transwell assay to evaluate the invasion and migration of the cells, and found that overexpression of SH3PXD2A-AS1 inhibited invasion and migration abilities (Fig. 2a), while knockout of this gene promoted invasion and migration of HTR8/SVneo cells (Fig. 2b). We also evaluated the effect of SH3PXD2A-AS1 on proliferation using the ethynyl deoxyuridine (EdU) assay and found that SH3PXD2A-AS1 overexpression inhibited proliferation of cells (Fig. 2c), while knockout exhibited opposite effects (Fig. 2d). These findings showed that SH3PXD2A-AS1 could restrain invasion, migration, and proliferation in trophoblast cells.

**Fig. 3:**
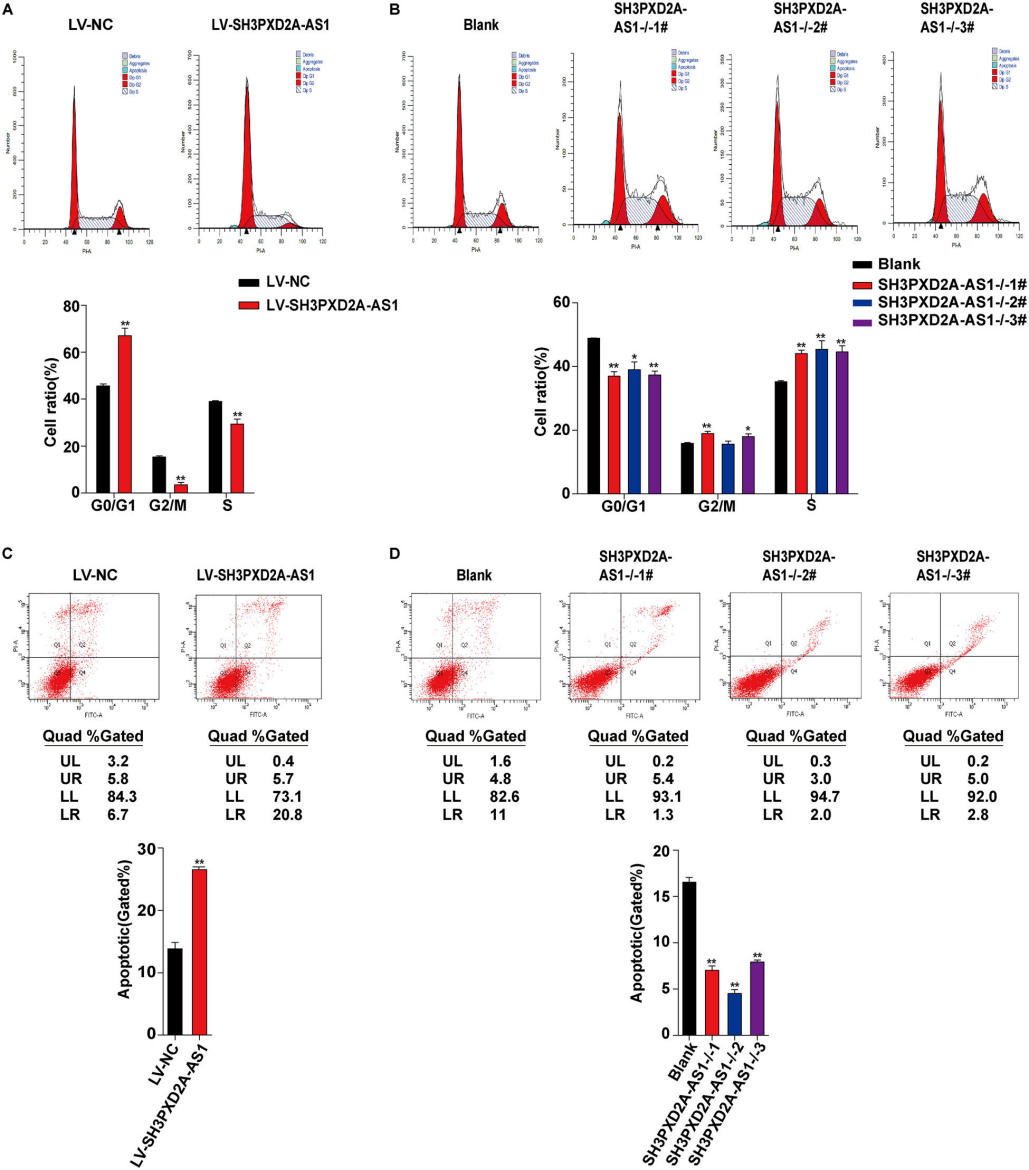
SH3PXD2A-AS1 arrests cell cycle and promotes death in HTR8/SVneo cells. **a** Cell cycle analysis by flow cytometry on cells transfected with lentivirus harboring human full-length SH3PXD2A-AS1 or empty vector. Upper panels: frequency histograms of cell cycle phases; lower panels: statistics of cell cycle phases. **b** Cell cycle analysis by flow cytometry on cells with SH3PXD2A-AS1 knockout. Upper panels: frequency histograms of cell cycle phases; lower panels: statistics of cell cycle phases. **c** Cell death analysis using Annexin-V assay and flow cytometry on cells with SH3PXD2A-AS1 overexpression. Upper panels: histograms of apoptotic cells; lower panels: statistics of apoptotic cells. **d** Cell death analysis using Annexin-V assay and flow cytometry on cells with SH3PXD2A-AS1 knockout. Upper panels: histograms of apoptotic cells; lower panels: statistics of apoptotic cells. **p < 0.01, *p < 0.05.

We further assessed the effect of SH3PXD2A-AS1 on cell cycle and death. Consistent with the results on cell proliferation, overexpression of SH3PXD2A-AS1 arrested cells in G0/G1 phase and reduced the number of cells entering S phase (Fig. 3a), while knockout promoted the transition from G0/G1 phase to S phase (Fig. 3b). Moreover, we also found that overexpression promoted cell death (Fig. 3c), while knockout inhibited cell death (Fig. 3d).

### CTCF as a downstream effector of SH3PXD2A-AS1

**Fig. 4:**
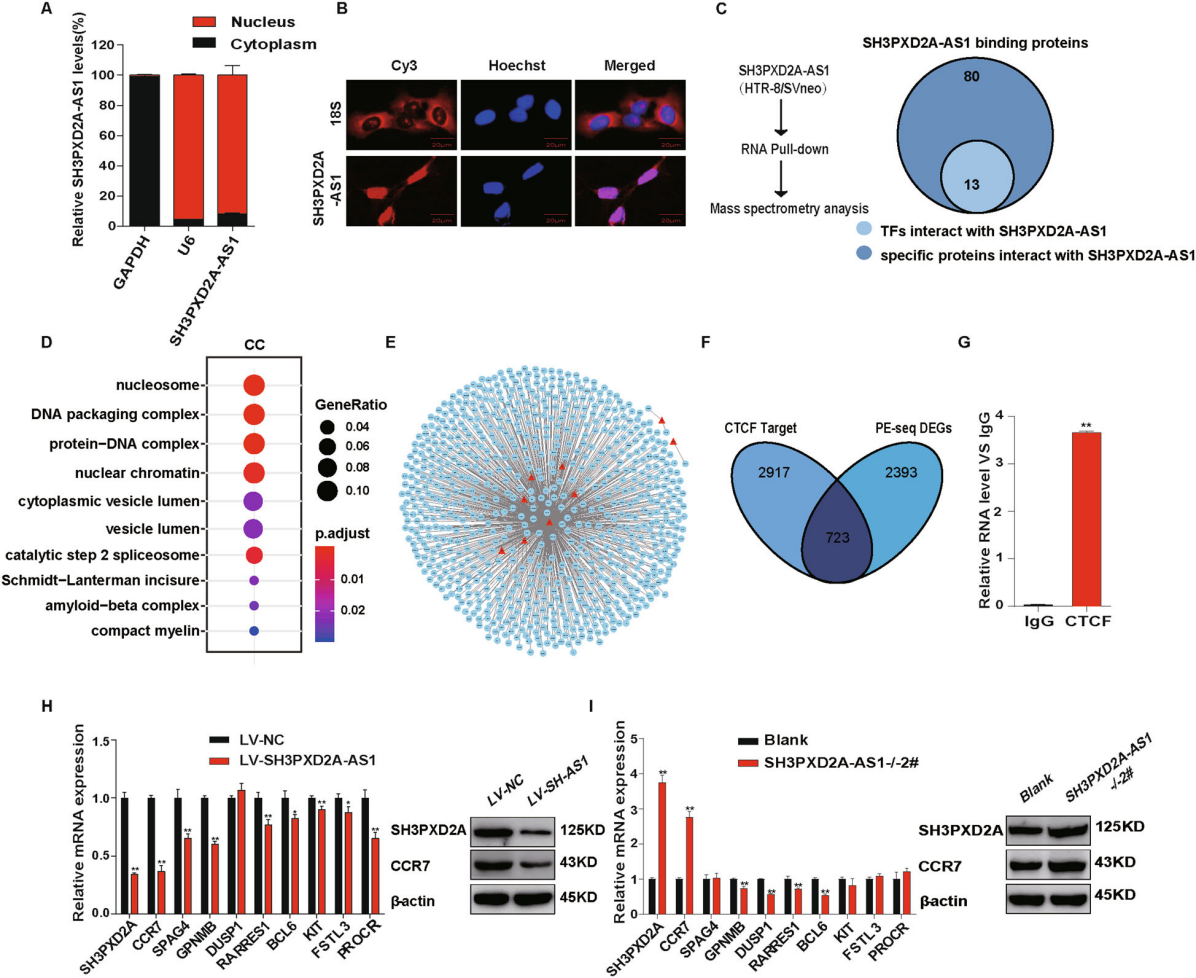
SH3PXD2A-AS1 binds to transcription factors and regulates genes potentially involved in EOSPE. **a** The subcellular localization of SH3PXD2A-AS1 in HTR-8/SVneo cell detected using cell fractionation. U6: nucleus marker; GAPDH: cytoplasm marker. **b** The subcellular localization of SH3PXD2A-AS1 in HTR-8/SVneo detected using FISH. Red: SH3PXD2A-AS1; blue: nucleus. **c** Proteins interacting with SH3PXD2A-AS1 detected in HTR8/SVneo cell by pulldown and mass spectrometry. **d** GO terms (Cellular Components) with over-presentation of SH3PXD2A-AS1-binding proteins. **e** TF-target network for DEG of EOSPE. Red triangles represent transcription factors (TFs) binding SH3PXD2A-AS1; blue circles represent differentially expressed target genes in EOSPE; **f** overlaps between CTCF target genes (3640) curated in database and differentially expressed genes (3116) detected in the transcriptome sequencing on placentae of patients with EOSPE. **g** Binding proteins of SH3PXD2A-AS1 detected by RIP assays and qRT-PCR. **h** SH3PXD2A-AS1 overexpression reduces CTCF target genes. Top 10 target genes with highest expression change in transcriptomes of EOSPE patients were evaluated using qRT-PCR. Left histogram blot shows the results of qRT-PCR. The reduced expression of two targets were verified by western blot (left). **i** SH3PXD2A-AS1 knockout increases CTCF target genes. Top 10 target genes with highest expression change in transcriptomes of EOSPE patients were evaluated using qRT-PCR. Left histogram blot shows the results of qRT-PCR. The increased expression of two targets were verified by western blot (left). **p < 0.01, *p < 0.05.

Using subcellular fractionation, we detected ~92% of SH3PXD2A-AS1 in the nucleus, and 8% of SH3PXD2A-AS1 in the cytoplasm (Fig. 4a). The nuclear localization of SH3PXD2A-AS1 was confirmed by fluorescence in situ hybridization (FISH) assay (Fig. 4b), suggesting that SH3PXD2A-AS1 might participate in transcriptional regulation. In order to search for SH3PXD2A-AS1-binding TFs, we performed RNA pulldown and mass spectrometry (Fig. S2A), and identified 93 proteins binding to SH3PXD2A-AS1, including 13 TFs (Fig. 4c, Fig. S2B, Table S4). The SH3PXD2A-AS1-binding proteins are enriched for nuclear components of gene ontology (GO) terms, such as nucleosome and chromatin (Fig. 4d).

By searching databases TRED^40^, ITFP^41^, ENCODE^42^, Neph2012^43^, TRRUST2^44^, and Marbach2016^45^ (Table S5), we obtained 3836 targets of these 13 TFs (Fig. S2C, D,Table S5). Of the 3836 target genes, 758 are differentially expressed genes (DEGs) in placentae of EOSPE patients. We extracted TF-target regulatory network for the EOSPE DEGs (Fig. 4e). The transcription factor CTCF has the highest betweenness centrality (Table S5), suggesting that CTCF might play central role in this regulatory network. CTCF targets 3640 genes and about 19.8% (723/3640) are differentially expressed in EOSPE. These targets account for about 23% (723/3116) of the differentially expressed genes (DEGs) (Fig. 4f).

We performed RNA immunoprecipitation (RIP) and qRT-PCR to confirm the interaction of SH3PXD2A-AS1 with CTCF (Fig. 4g). Although CTCF is universally expressed, its activity in placenta might be regulated by SH3PXD2A-AS1. We detected no significant differences of CTCF expression in HTR-8/SVneo cells with overexpression and knockout of SH3PXD2A-AS1 (Fig. S2E), suggesting that the expression level of CTCF is not regulated by SH3PXD2A-AS1. We suggested that upregulated SH3PXD2A-AS1 in EOSPE might cause gene dysregulation through binding to CTCF. To validate this hypothesis, we performed SH3PXD2A-AS1 overexpression or knockout in trophoblast cells and detected expression levels of top 10 EOSPE-upregulated CTCF target genes using qRT-PCR and western blotting assays. We found that these CTCF targets were indeed regulated by SH3PXD2A-AS1 (Fig. 4h, i). Among these CTCF targets, SH3PXD2A and CCR7 showed the most significant expression changes at both mRNA level and protein level in cells with overexpression or knockout of SH3PXD2A-AS1 (Fig. 4h, i). The discrepancies about the expression changes of the target genes (Fig. 4h, i) suggest that some other TFs or factors may be involved. Since SH3PXD2A or CCR7 are reported to be involved cancer cell invasion and migration^46,47^, downregulation of CTCF targets SH3PXD2A and CCR7 by SH3PXD2A-AS1 might have similar functional effects on trophoblast cells during placentation.

### SH3PXD2A-AS1 inhibits the expression of SH3PXD2A and CCR7 through recruiting CTCF to the promoters

**Fig. 5:**
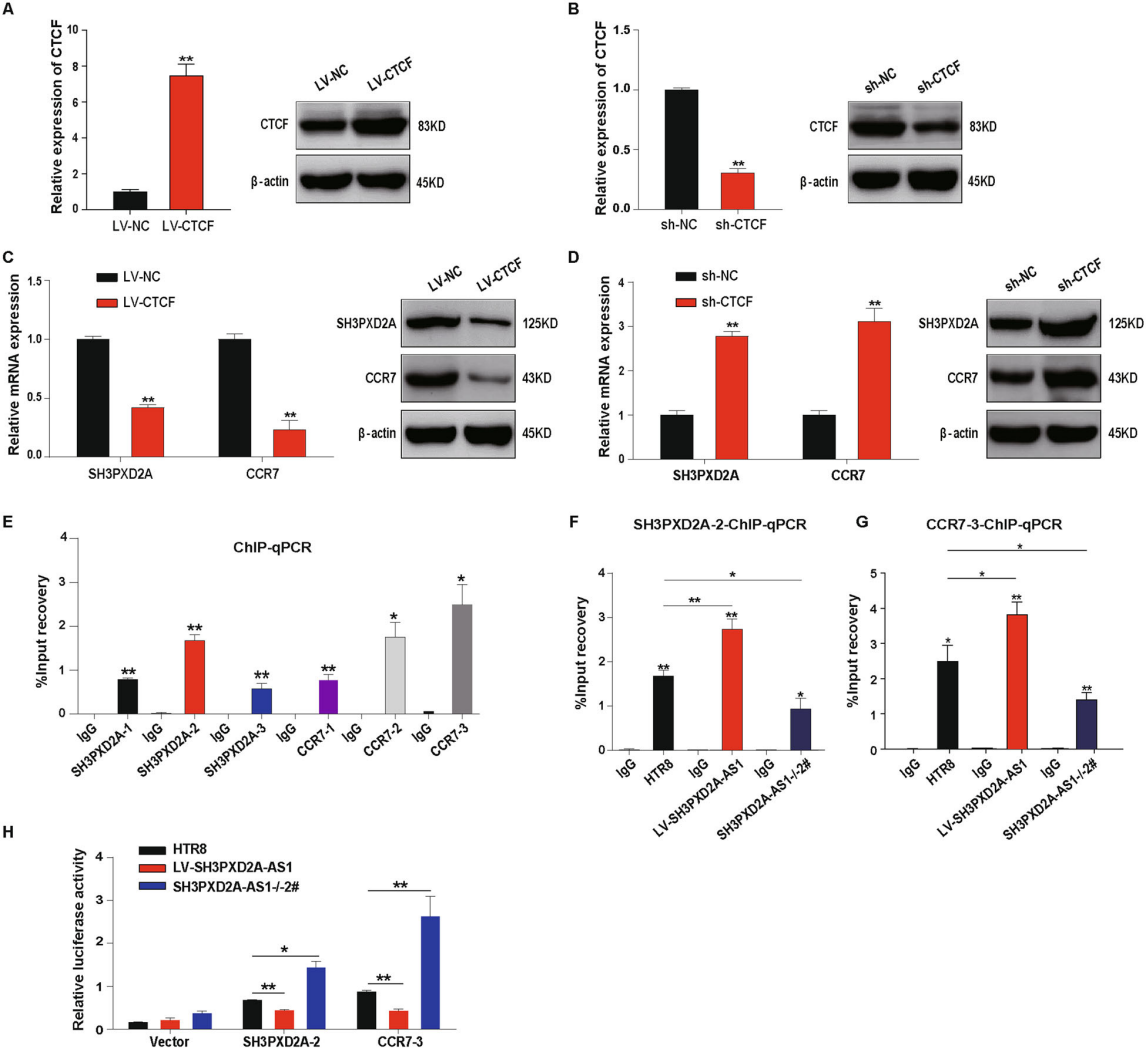
SH3PXD2A-AS1 recruits CTCF to the promoters of SH3PXD2A and CCR7 and inhibits their transcription. **a** The expression level of CTCF in HTR8/SVneo cells transfected with full-length CTCF. Left: results of qPCR; right: results of western blot. **b** The expression level of CTCF in HTR8/SVneo cells transfected with shRNA of CTCF. Left: results of qPCR; right: results of western blot. **c** The reduced expression of SH3PXD2A and CCR7 in HTR8/SVneo cells with CTCF overexpression. Left: results of qPCR; right: results of western blot. **d** The increased expression of SH3PXD2A and CCR7 in HTR8/SVneo cells with shRNA of CTCF. Left: results of qPCR; right: results of western blot. **e** CTCF’s binding to the promoters of SH3PXD2A and CCR7 detected by ChIP assays and qPCR in HTR8/SVneo cells. **f** CTCF’s binding to the promoter of SH3PXD2A-2 detected by ChIP assays with SH3PXD2A-AS1 overexpression or knockout in HTR8/SVneo cells. **g** CTCF’s binding to the promoter of CCR7-3 detected in HTR8/SVneo cells with SH3PXD2A-AS1 overexpression or knockout using ChIP assays. **h** The transcription activity of SH3PXD2A-2 and CCR7-3 promoters detected in HTR8/SVneo cells with SH3PXD2A-AS1 overexpression or knockout using luciferase reporter assay. **p < 0.01, *p < 0.05.

In order to confirm the effect of CTCF on SH3PXD2A and CCR7 expression, we performed the overexpression (Fig. 5a) and knockdown (Fig. 5b) of CTCF in HTR-8/SVneo cells. We found significant downregulation of SH3PXD2A and CCR7 at both mRNA level and protein level in cells with overexpression of CTCF (Fig. 5c) and upregulation of SH3PXD2A and CCR7 in cells with knockdown of CTCF (Fig. 5d). The interaction we detected between SH3PXD2A-AS1 and CTCF (Fig. 4g) suggested that this lncRNA may recruit CTCF to the promoters of SH3PXD2A and CCR7. We confirmed the interaction between CTCF and the promoter regions of SH3PXD2A and CCR7 using chromatin immunoprecipitation (ChIP) assays (Fig. 5e). Moreover, SH3PXD2A-AS1 overexpression promoted the recruition of CTCF to the promoters of SH3PXD2A and CCR7, while knockout exhibited opposite effects (Fig. 5f, g).

We further tested the effect of SH3PXD2A-AS1 on the transcriptional activity of the promoters of SH3PXD2A and CCR7 using luciferase as a reporter, and found that SH3PXD2A-AS1 overexpression inhibited the transcription of the reporter gene, whereas its knockout promoted the transcription (Fig. 5h). These results suggested that SH3PXD2A-AS1 inhibit the expression of SH3PXD2A and CCR7 through recruiting CTCF to the promoter regions.

### SH3PXD2A-AS1 inhibits invasion and migration through downregulating SH3PXD2A and CCR7

**Fig. 6:**
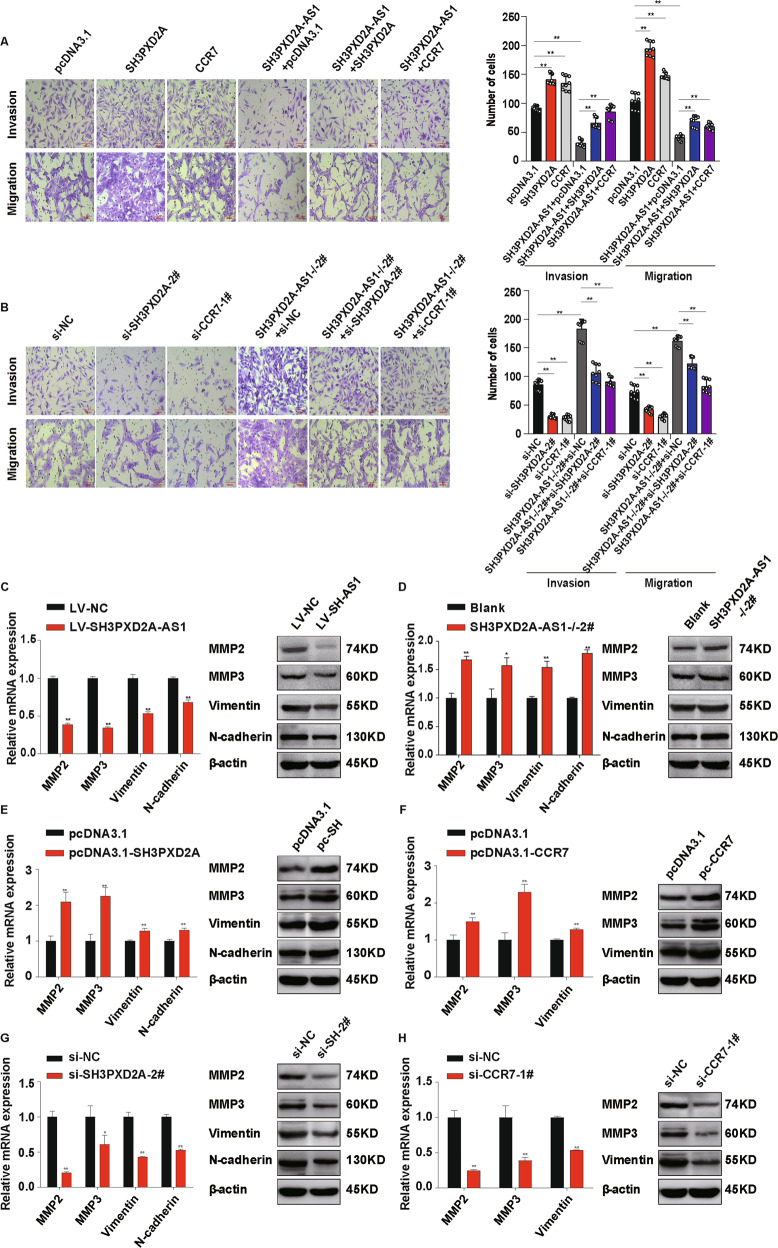
SH3PXD2A-AS1 inhibits the effects of SH3PXD2A and CCR7 on invasion and migration of trophoblast cells. **a** Overexpression of either SH3PXD2A or CCR7 partially rescues the invasion and migration abilities inhibited by overexpression SH3PXD2A-AS1 in HTR8/SVneo cells. Left: cells stained with crystal violet after Transwell assays; right: quantification of cells with invasion or migration for Transwell assays. **b** Knockout of SH3PXD2A-AS1 partially rescues the invasion and migration abilities inhibited by either knockdown of SH3PXD2A or CCR7 in HTR8/SVneo cells. Left: cells stained with crystal violet after Transwell Assays; Right: quantification of cells with invasion or migration for Transwell assays. **c** SH3PXD2A-AS1 overexpression inhibited the expression of marker genes of invasion and migration in HTR8/SVneo cells. Left: results of qPCR; right: results of western blot. **d** SH3PXD2A-AS1 knockout promoted the expression of marker genes of invasion and migration in HTR8/SVneo cells. Left: results of qPCR; right: results of western blot. **e** The upregulation of marker genes of invasion and migration in cells with SH3PXD2A overexpression. Left: results of qPCR; right: results of western blot. **f** The upregulation of marker genes of invasion and migration in cells with CCR7 overexpression. Left: results of qPCR; right: results of western blot. **g** The downregulation of marker genes of invasion and migration in cells with SH3PXD2A knockdown. Left: results of qPCR; right: results of western blot. **h** The downregulation of marker genes of invasion and migration in cells with CCR7 knockdown. Left: results of qPCR; right: results of western blot. **p < 0.01, *p < 0.05.

To further confirm that SH3PXD2A-AS1 regulates the transcription of SH3PXD2A and CCR7 through CTCF, we established overexpression and/or knockdown of SH3PXD2A and CCR7 in different combination in HTR-8/SVneo cells (Fig. 6a, b, Fig. S3A, B). SH3PXD2A-AS1 overexpression inhibited invasion and migration in HTR-8/SVneo cells, while overexpression of SH3PXD2A or CCR7 promoted invasion and migration (Fig. 6a). Overexpression of SH3PXD2A or CCR7 partially rescued the invasion and migration that was inhibited by SH3PXD2A-AS1 overexpression (Fig. 6a). On the contrary, SH3PXD2A-AS1 knockout promoted invasion and migration of the trophoblast cells, whereas knockdown of SH3PXD2A or CCR7 could inhibited invasion and migration (Fig. 6b). Knockout of SH3PXD2A-AS1 could partially reduce the inhibition on invasion and migration by SH3PXD2A or CCR7 knockdown (Fig. 6b).

Since MMP2, MMP3, Vimentin, and N-cadherin are well-established markers for invasion and migration in cancer^48–50^. We found that SH3PXD2A-AS1 overexpression inhibited the expression of these markers at both mRNA and protein level in HTR8/SVneo cells, while knockout exhibited opposite effects (Fig. 6c, d). Moreover, we detected significant upregulation of these markers in HTR8/SVneo cells with overexpression of SH3PXD2A or CCR7 using qRT-PCR and western blotting (Fig. 6e, f), and downregulation of these markers in cells with knockdown of these two genes (Fig. 6g, h). The results on these markers supported our findings on effects of the regulation of SH3PXD2A and CCR7 by SH3PXD2A-AS1 in the invasion and migration of trophoblast cells.

The results of RNA-seq data show that SH3PXD2A and CCR7 were upregulated in the EOSPE placentae, and CTCF showed no change (Fig. S4A). We calculated the Pearson correlation coefficient between the expression level of SH3PXD2A-AS1 and the other three genes, and found that SH3PXD2A and CCR7 are positively correlated to the expression of SH3PXD2A-AS1. But there was no correlation between the expression of CTCF and SH3PXD2A-AS1 (Fig. S4B). We got the similar results on the placentae from 20 PE patients and 20 normal subjects using qRT-PCR (Fig. S4C, D). Since SH3PXD2A and CCR7 promoted invasion and migration of trophoblasts (Fig. 6), we speculate that the upregulation SH3PXD2A and CCR7 in the placentae of the PE patients might be due to complex compensatory mechanisms. The immunohistochemistry on placentae showed that SH3PXD2A, CCR7, and CTCF were mainly expressed in the membrane of syncytiotrophoblast and vascular endothelial cells, less in cytotrophoblasts cells (Fig. S4E).

Taken together, our findings demonstrate that SH3PXD2A-AS1 recruits the transcription suppressor CTCF to the promoter regions of SH3PXD2A and CCR7 and inhibits the transcription of these two important factors for invasion and migration (Fig. S5). The identified pathway “SH3PXD2A-AS1-CTCF-SH3PXD2A/CCR7” in trophoblast invasion and migration may be crucial for placentation. The disturbance of this pathway may lead to poor placentation, which is considered to be important in the development of PE.

## Discussion

It is widely accepted that PE may have placental origin^16^. Recent studies suggest that dysregulation of some placental lncRNAs may be involved in the development of PE^51^. Some lncRNAs show exclusive or predominant expression in placenta and exhibit altered expression pattern in placentae from complicated pregnancies which may be epigenetically regulated in the placental tissues^52^. For example, H19 is one of highly expressed gene in placenta^53^, and is reported to be downregulated in the placentae of patients with early-onset preeclampsia^54^. The altered imprinting of the genomic region covering H19 and its adjacent gene IGF2 in human placentae is associated with PE^55^. H19 inhibits the proliferation of trophoblast cells through encoding miR-675, which down-regulates the expression of NOMO1^54^. SNHG5 is known to promote cancer cell proliferation and survival^56^, and is recently reported to promote the proliferaction, invasion, and migration of trophoblast cells^57^. TUG1 is well documented to promote the proliferation, invasion, and colony formation of cancer cells^58^ and trophoblast cells as well^59^. Some other lncRNAs, such as MEG3 and MALAT-1, are reported to be involved in the invasion and migration of trophoblast cells^28,30^. These studies show that numerous lncRNAs have been found involved in invasion and migration of trophoblast cells, which are crucial for placenta development. A systematic search for preeclapsia-associated lncRNAs followed by in-depth studies on their functions is still in urgent need for understanding the pregnancy complications with placenta origin such as preelampsia.

We have carried out a transcriptome sequencing on the placentae from EOSPE patients and normal controls, and found 383 differentially expressed lnRNAs. SH3PXD2A-AS1 is among the top upregulated lncRNAs (Fig. 1a). It has a length of 1066 bases and is an antisense transcript of the gene SH3PXD2A. We checked the expression level of SH3PXD2A-AS1 in placentae from PE patients and controls, it was significantly upregulated in both EOSPE and LOSPE patients (Fig. 1b). Of the 20 patients, 80% (8/10) in EOSPE or LOSPE showed increased placental expression of SH3PXD2A-AS1 compared to the controls (Fig. 1c), and the expression level was positively correlated with blood pressure (Fig. 1d, e). Since lncRNAs often carry out their functions through interacting with proteins, we searched for potential lncRNA-protein interactions from databases and predictions and obtained 551 potential SH3PXD2A-AS1–protein interactions, including 218 TFs (Fig. 1f). We demonstrated that SH3PXD2A-AS1 suppressed migration, invasion and proliferation and promoted cell death in trophoblast cells by overexpression or knockout of SH3PXD2A-AS1 (Figs. 2, 3). These findings suggest that SH3PXD2A-AS1 may be involved in the progression of PE through prohibiting the function of trophoblast cells.

SH3PXD2A-AS1 was mostly located in nucleus (Fig. 4a, b). Of the 93 SH3PXD2A-AS1-binding proteins we detected using RNA pulldown and mass spectrometry, including 13 transcription factors and some other nuclear proteins (Fig. 4c). CTCF is one of the 13 transcription factors binding to SH3PXD2A-AS1, which has 3640 target genes, and 19.8% of the target genes are differentially expressed in EOSPE (Fig. 4f, g), suggesting that SH3PXD2A-AS1 may be involved in the pathogenesis of PE by recruiting CTCF to a large number of PE-associated genes. CTCF is a conserved nucleoprotein with 11 zinc finger structures, which can regulate functions through transcription activation, inhibition and gene imprinting^60^. Some lncRNAs are known to bind CTCF. For example, lncRNA MYCNOS upregulates MYCN expression in neuroblastoma by recruiting CTCF to promoter region of this gene to induce chromatin remodeling^61^. LncRNA FOXD3-AS1 interacts with PAPR1 to facilitate the binding of CTCF to the promoters of tumor-suppressive genes^62^. In this study, we found that, among the top 10 differentially expressed CTCF-binding proteins in EOSPE patients, SH3PXD2A and CCR7 were downregulated by lncRNA SH3PXD2A-AS1 in trophoblast cells (Fig. 4h,i). Furthermore, we confirmed that SH3PXD2A-AS1 promoted the recruition of CTCF to SH3PXD2A and CCR7 promoters, and inhibited the transcription of these two genes using ChIP and luciferase reporter assays (Fig. 5e–h). These results strongly suggest that “SH3PXD2A-AS1–CTCF–SH3PXD2A/CCR7” might be a potential pathway in regulating the trophoblast proliferation, invasion, and migration.

A number of studies have shown that insufficient invasion of placental trophoblast cells leads to obstruction of uterine spiral artery remodeling, placental ischemia and hypoxia, resulting in impaired placental function and the occurrence of PE^2,63^. SH3PXD2A, also known as Tks5, is a tyrosine kinase substrate containing five SH3 domains. It promotes the tumor metastasis and epithelial-mesenchymal transformation, and participates in various physiological and pathological processes of cancer^64,65^. And Chemokine receptor 7 (CCR7) is a G protein-coupled receptor containing 7 transmembrane domains, regulating cell function and immune balance. CCR7 is highly expressed in villous and cancers, affecting the migration and invasion of trophoblast and tumor cells^66–68^. We found that SH3PXD2A-AS1 inhibited invasion and migration of HTR-8/SVneo cell, whereas SH3PXD2A and CCR7 were able to partially rescue the invasion and migration inhibited by SH3PXD2A-AS1 (Fig. 6).

We observed upregulation of SH3PXD2A-AS1 in the placentae of PE patients (Fig. 1b, c), but we found that SH3PXD2A and CCR7 were upregulated in peeclamptic placentae (Fig. S4A, C), probably due to the compensation to the deficient trophoblast functions. In previous studies^69,70^, SH3PXD2A and CCR7 were detected to be upregulated in the placentae of PE patients. Consistent with our results on the effect of CCR7 and SH3PXD2A, a recent study found that CCR7 promotes migration and invasion in trophoblast cells^68^, and that SH3PXD2A promotes the migration and invasion in tumor cells^64,65^. It is widely accepted that insufficient trophoblast invasion and impaired spiral artery remodeling can result in defective placentation, leading to ischemia and hypoxia^1^. It is reported that low oxygen supply in placenta could increase the expression of CCR7^71^ and SH3PXD2A^69^ in PE, which might constitute a compensatory mechanism to ensure proper pregnancy progress.

However, we do not know the molecular mechanism for compensatory upregulation of these two genes. A previous study suggests that the upregulation of SH3PXD2A in PE placentas may be caused by the hypermethylated CGI34 in the gene body of SH3PXD2A^70^. The methylation in gene body CGI is positively correlated with gene expression^72^. CCR7 is an important regulator for immune inflammatory response in lymphoid organs^73,74^ and it may contribute to the regulation of inflammatory responses through immune mechanisms during healthy pregnancy^75,76^. Therefore, SH3PXD2A and CCR7 in the placenta may be regulated by multiple mechanisms, and our in vitro study focused on their regulation by SH3PXD2A-AS1 to inhibit the invasion and migration in trophoblast cells.

In conclusion, from the differentially expressed lncRNAs in the placental transcriptomes of EOSPE patients, we have identified SH3PXD2A-AS1 as a potential causal factor for this disease. The upregulation of SH3PXD2A-AS1 in placenta is positively correlated with clinical severity of PE. By computational and experimental search for its interacting proteins, we have further identified CTCF as a key TF recruited by SH3PXD2A-AS1 to regulate the downstream targets. Then we further established “SH3PXD2A-AS1-CTCF-SH3PXD2A/CCR7” pathway in regulating the invasion and migration of trophoblast cells (Fig. S5). Our findings suggest that dysregulation of the lncRNA SH3PXD2A-AS1 may perturbate this pathway, leading to poor placentation and contributing to the pathogenesis of PE.

## Materials and methods

### Preeclampsia patients and placental tissue collection

This research has been approved by The Research Ethics Board of Nanfang Hospital, Southern Medical University, China and all patients have signed informed consent. All samples were collected at Department of Obstetrics & Gynecology of Nanfang Hospital. The 41 placental samples for transcriptome sequencing were collected from January 2015 to July 2016, and 40 placental samples for qRT-PCR were collected from December 2016 to May 2018. The placental tissues were collected from the mid-section between the chorionic and maternal basal surfaces at four different positions of the placenta within 5 min after delivery. The tissues were washed immediately with PBS buffer, preserved in RNA later at −80 °C for later RNA isolation. The clinical characteristics of each patient were extracted from the medical records of clinical diagnosis, which strictly followed the American Board of Obstetrics and Gynecology, Williams Obstetrics 24th edition.

### Identifying lncRNA SH3PXD2A-AS1 as potential PE-causal gene from transcriptome sequencing data

We have carried out transcriptome sequencing and identified a total number of 3116 differentially expressed genes, including 383 differentially expressed lncRNAs. SH3PXD2A-AS1 was among the top DElncRNAs based on fold change. From DElncRNA–protein interaction data, we obtained SH3PXD2A-AS1 218 interacting TFs which target 10976 genes. Because these targets are enriched with DEGs in EOSPE, we chose SH3PXD2A-AS1 as potential PE-causal gene for further study.

### RNA isolation and quantitative real-time PCR

Total RNA was isolated using the RNeasy Plus Universal Mini Kit (Qiagen) according to the manufacturer’s instruction. The RNAs (500 ng) were reverse transcribed using PrimeScriptTMRT reagent Kit (Takara, Japan), and qRT-PCR was performed with SYBR Premix Ex TaqTM kit (Takara, Japan) in a LightCycler 480 (Roche, Swiss) system to detect expression of genes, following the manufacturer’s instruction. The sequences of specific primers were shown in Table S6. The relative gene expression was calculated using 2^−▵▵▵CT^ method and converted to fold changes using ACTB as internal controls.

### Cell culture

HTR8/SVneo cell line was obtained from American Type Culture Collection (Manassas, USA), and was cultured in RPMI 1640 medium (Corning, USA) supplemented with 10% fetal bovine serum (Gibco, USA) in humidified air at 37 °C with 5% CO_2_.

### Lentiviral expression constructs and transfection

The full length of lncRNA SH3PXD2A-AS1 and CTCF were synthesized and cloned into pGC-FU vector (Genechem, China). The shRNAs targeting CTCF were designed and cloned into pGC-FU vector (Genechem, China). HTR8/SVneo cells were transfected these plasmids following the manufacturer’s instruction and stable cell lines were selected using puromycin (Gibco, USA). The interference sequence of sh-CTCF was gcCTCTTTCTTGGCAAAGTTT.

### Plasmid and siRNA transfection

The plasmids pcDNA3.1-SH3PXD2A, pcDNA3.1-CCR7, si-SH3PXD2A and si-CCR7 were purchased from Genechem (Genechem, China). The transfection was done using Lipofectamine 3000 (Invitrogen) according to the manufacturer’s instruction. After 48 h transfection, HTR8/SVneo cells were harvested for further experiments. The sequences of siRNAs were listed in Table S6.

### CRISPR/Cas9-mediated SH3PXD2A-AS1 knockout

HTR8/SVneo cells were transfected with Cas9 expression plasmid, guide RNA plasmid (designed online at Crispr.Mit.Edu), and a donor plasmid containing the Puc57-puro insert. After selection with puromycin for a week, HTR8/SVneo cells were plated at 96-well plates to select monoclonal cells for further experiments. The sequences of specific primers were shown in Table S6.

### Transwell assays

The 24-well Transwell chambers with 8-μm pore size polycarbonate membranes (Corning, USA) were used to test invasion and migration of HTR8/SVneo cells. The 200 ul cell suspension without FBS was added in the upper side of the membrane coated with or without Matrigel (BD, USA), and 600 μl culture medium RPMI 1640 containing 10% FBS was added in the lower chamber and placed in the cell culture incubator. After 24 h (for migration) and 48 h (for invasion), the upper chambers were removed with cottons swabs. The lower chambers were fixed by methanol for 30 min and stained with 0.1% crystal violet solution for 20 min. After the chambers were washed with PBS 3 times, the numbers of cells that have penetrated the filter membrane were observed under optical microscope.

### EdU assays

The cell proliferation was determined by EdU assays using the 5-ethynyl-2′-deoxyuridine labeling/detection kit (Ribobio, China). Each confocal dish inoculated with cells was added with 200 μl diluent A and incubated in the cell incubator for 2 h. Cells were washed with PBS buffer, and then fixed by 4% paraformaldehyde for 30 min and decolorized with 200 μl of 2 mg/ml glycine for 5 min in a shaker at room temperature. Then, 200 μl of 0.5% Triton X-100 was added to each well and put at room temperature for 10 min. After washed once with PBS buffer, each well was incubated with 200 μl of pre-prepared 1× Apollo stain at room temperature and incubated in the dark for 30 min. After washing twice with PBS buffer, 0.5% Triton X-100 was added to each well and incubated for 10 min. Each well was stained with 200 μl diluent F, and incubated in the dark at room temperature for 30 min, and then washed twice with PBS buffer. After the staining, the dishes were observed under fluorescence microscope.

### Flow cytometric analysis of cell cycle and cell death

Cell cycle was evaluated using the RNase A/Propidium Iodide Detection kit (KeyGEN, China). The staining buffer of PI/RNase A was prepared at the proportion was 9:1. After precipitated by centrifugation, the cell pellets were suspended using 500 μl of 70% cold ethanol, and then fixed for 2 h to overnight at 4 °C. After washing with PBS before staining, 500 μl of precooled PI/RNase A staining solution was added, mixed, and reacted at room temperature to avoid light for 30–60 min. The percentage of G0/G1, S, and G2/M phases were estimated using flow cytometry (BD LSRFortessa, USA). Cell death was determined using the Annexin-V/Propidium Iodide Detection kit (KeyGEN, China). The detached cells in the culture medium were collected first, and the attached cells were collected after trypsin digestion. The cells were precipitated using centrifugation and resuspended with 500 μl Binding Buffer. 5 μl of Annexin V-FITC and 5 μl of propidium iodide (PI) was added and mixed gently. The reaction was conducted in the dark at room temperature for 5–15 min, and the percentage of early, late apoptosis, viable and dead cells was detected by flow cytometry (BD LSRFortessa, USA) within 1 h.

### RNA pulldown and mass spectrometry assay

SH3PXD2A-AS1 and its antisense RNA were made from transcription with the Biotin RNA Labeling Mix (Roche, USA) and T7 RNA polymerase (Roche, USA). Biotinylated RNA was incubated with HTR8/SVneo cell nuclear extracts, and pulldown proteins were run on SDS-PAGE gels (Sigma, USA) and stained with silver staining solution (Beyotime, China), followed by Mass spectrometry.

### Subcellular fractionation

Cytosolic and nuclear fractions of HTR8/SVneo cell were prepared using PARIS Kit (Life Technologies, USA) following the manufacturer’s instruction. The levels of SH3PXD2A-AS1, GAPDH, and U6 were examined by qRT-PCR. GAPDH was used as cytoplasm control and U6 as nuclear control.

### Fluorescence in situ hybridization (FISH)

The probe of SH3PXD2A-AS1 was made by RiboBio (RiboBio, China). The localization and distribution of lncRNA SH3PXD2A-AS1 were detected using Fluorescence In Situ Hybridization Kit (RiboBio, China) following the manufacturer’s instruction.

### RIP assays

RNA pulldown experiments were carried out using EZ-Magna RIP Kit (Millipore, USA) following the manufacturer’s instruction. HTR-8/SVneo cells were collected by centrifugation, and the cell pellets were then lysed in RIP lysis buffer. Anti-CTCF antibodies and normal lgG (Millipore, USA) were used for immunoprecipitation, and the immunoprecipitated RNA was analyzed by qRT-PCR. The sequences of specific primers were listed in Table S6.

### Western blot assays

Total cellular proteins were extracted using Whole Protein Extraction Kit (KeyGEN, China). Protein concentration was determined by BCA Protein Assay Kit (KeyGEN, China). The following primary antibodies were used: anti-Fish, anti-CCR7, anti-CTCF (Abcam, UK), anti-MMP2, anti-MMP3, anti-Vimentin, anti-N-cadherin (Cell Signaling Technology, USA), and anti-β-Actin (Cell Signaling Technology, USA).

### **C**hIP assays

ChIP assays were performed using the Agarose ChIP Kit (Pierce, USA) following the manufacturer’s instruction. Anti-CTCF was purchased from Abcam (Abcam, UK). Immunoprecipitated DNA was analyzed using qRT-PCR. The binding sites of SH3PXD2A and CCR7 promoter were predicted using GRCh38/hg38 version of the genome database at UCSC genome browser. The binding sites and primer sequences of gene promoters were listed in Table S7.

### Luciferase reporter assays

We cloned the SH3PXD2A site 2 and CCR7 site 3 (Table S7), which bind to CTCF with strongest signal, into PGL3-Basic vector. The empty plasmid vector pRL-TK was used as control. The promoter activity was evaluated using Luciferase Assay Kit (Promega, USA) following the manufacturer’s instruction. After incubated for 48 h, the cells were lysed in 1× Passive lysis and examined for luciferase activity, and the Renilla luciferase was used as a control.

### Immunohistochemistry

Placental samples were fixed in 4% paraformaldehyde, and then treated with dimethylbenzene and alcohol. The sections were incubated in citric acid antigen retrieval buffer and heated in a microwave. After washing in PBS for three times, the tissue slides were incubated in 3% H_2_O_2_ for 20 min. Then the sections were immersed in 3% BSA for 1 h at room temperature. The sections were then incubated with the rabbit anti-SH3PXD2A antibody (1:200; Proteintech, China), rabbit anti-CCR7 antibody (1:200; Abcam, UK) and rabbit anti-CTCF antibody (1:100; Abcam, UK) overnight at 4 °C. After that, the samples were incubated with a goat anti-rabbit secondary antibody (1:2000; Abcam, UK) for 1 h at room temperature. After washing in PBS, the sections were subjected to the chromogenic reactions with diaminobenzidine for 5 min and hematoxylin for 2 min. Photos were taken at ×200 under a microscope (Olympus, Tokyo, Japan).

### Quantification and statistical analysis

All statistical analyses were performed using SPSS20.0 (IBM), and the data were presented as the mean ± S.D of three independent experiments. The comparison between the two independent groups was conducted using Student’s *t*-test or Mann–Whitney test. Chi-squared test was used to analysis frequencies and correlation analyses were assessed using the Pearson correlation coefficient. *P* value less than 0.05 was considered statistically significant.

## Supplementary information

Supplementary Information

Supplementary Figure S1

Supplementary Figure S2

Supplementary Figure S3

Supplementary Figure S4

Supplementary Figure S5

Supplementary Table S1

Supplementary Table S2

Supplementary Table S3

Supplementary Table S4

Supplementary Table S5

Supplementary Table S6

Supplementary Table S7
