# Supplementary material for: Association of lncRNA SH3PXD2A-AS1 with preeclampsia and its function in invasion and migration of placental trophoblast cells
Source: Cell Death Dis. 2020 Jul 27;11(7):583. doi: 10.1038/s41419-020-02796-0 (PMC7385659; doi:10.1038/s41419-020-02796-0)
Supplement: Supplementary file 1 — Supplementary Information [file 41419_2020_2796_MOESM1_ESM.docx]

**Supplemental Figure Legends**

**Figure S1. Detection of the expression levels of genes in cells with overexpression or knockout of SH3PXD2A-AS1.**

(A) The qRT-PCR quantification of SH3PXD2A-AS1 expression in HTR8/SVneo cells transfected with full-length sequence of human SH3PXD2A-AS1 and empty vector.

(B) The qRT-PCR quantification of SH3PXD2A-AS1 expression in HTR8/SVneo cells with/without SH3PXD2A-AS1 knockout.

***p* < 0.01, **p* < 0.05.

**Figure S2. SH3PXD2A-AS1-TF-target network.**

(A) The silvery staining of the gel with the proteins pulled down by SH3PXD2A-AS1 and antisense RNA.

(B) SH3PXD2A-AS1-protein interaction network. Red nodes: SH3PXD2A-AS1; blue triangles represent the transcription factors; blue circles represent the other proteins.

(C) TF-target network for DEG of EOSPE. Red triangles represent transcription factors (TFs) binding SH3PXD2A-AS1; Blue circles represent target genes of the transcription factors.

(D) The overlapping genes between the targets of SH3PXD2A-AS1 binding transcript factors (TFs) and differentially expressed genes (DEGs) in EOSPE.

(E) The expression of CTCF detected by qRT-PCR and western blotting in HTR8/SVneo cells with SH3PXD2A-AS1 overexpression and knockout.

***p* < 0.01, **p* < 0.05, NS, not significant.

**Figure S3. The overexpression or knockdown of SH3PXD2A or CCR7 in HTR-8/SVneo.**

(A) The expression of SH3PXD2A and CCR7 detected by qRT-PCR and western blotting in HTR8/SVneo cells with transfection with pcDNA3.1- SH3PXD2A or pcDNA3.1- CCR7.

(B) The expression of SH3PXD2A and CCR7 detected by qRT-PCR and western blotting in HTR8/SVneo cells transfected with siRNAs.

***p* < 0.01, **p* < 0.05.

**Figure S4. The placental expression levels of SH3PXD2A-AS1, SH3PXD2A, CCR7 and CTCF in preeclampsia.**

(A) Boxplots of SH3PXD2A-AS1, SH3PXD2A, CCR7 and CTCF expression levels calculated from RNAseq data of EOSPE (n = 9) and normal placentae (n = 32). TPM: transcript per million.

(B) The correlation (Pearson correlation analysis) between the expression levels of SH3PXD2A-AS1 and SH3PXD2A, CCR7 or CTCF in RNAseq data. The *p* values are adjusted by FDR method.

(C) The expression levels of SH3PXD2A, CCR7 and CTCF measured by qRT-PCR, Y-axis: the fold-change; red bar: placentae of patients with PE, n=20; black bar: normal placentae, n=20.

(D) The correlation (Pearson correlation analysis) between the expression levels of SH3PXD2A-AS1 and SH3PXD2A, CCR7 or CTCF detected by qRT-PCRs in placentae from 20 PE patients and 20 normal subjects.

(E) SH3PXD2A, CCR7 and CTCF immunohistochemistry (IHC) in PE and normal placental tissues. The brownish particles showed the target proteins.

***p* < 0.01, **p* < 0.05.

**Figure S5. Schematic for illustrating potential role of SH3PXD2A-AS1 in etiology of preeclampsia.**

SH3PXD2A-AS1 may repress the transcription of SH3PXD2A and CCR7 through recruiting CTCF to the promoters and inhibit the invasion and migration of trophoblast cells.

**Supplemental Table Legends**

**Table S1: The differentially expressed lncRNAs in EOSPE.**

**Table S2: Clinical characteristics and relative expression level of SH3PXD2A-AS1.**

**Table S3: SH3PXD2A-AS1-TF-target hierarchical network.**

sheet1: SH3PXD2A-AS1-protein interactions collected from databases and predicted by catRAPID omics.

sheet2: SH3PXD2A-AS1-TF-target hierarchical network, including SH3PXD2A-AS1-TF interaction and TF-target regulation data.

shee3: Enrichment analysis was performed by clusterProfiler. The significant pathways (adjusted *p* value < 0.05) of DEGs of SH3PXD2A-AS1-TF targeting genes.

**Table S4: SH3PXD2A-AS1-binding proteins detected by Mass Spectrometry. The proteins in red are the transcription factors.**

**Table S5: The 3,836 targets of SH3PXD2A-AS1-binding TFs (RNA-pulldown) collected from databases.**

sheet1: TF-target network. TFs bind to SH3PXD2A-AS1 detected by RNA-pulldown.

sheet2: TF-DEGtarget network. These targets were differentially expressed in EOSPE.

sheet3: Betweenness centrality values of SH3PXD2A-AS1-binding TFs in TF-DEGtargets network.

shee4: The intersect between target of each TF and DEGs in EOSPE.

**Table S6: Sequences of primers and siRNAs.**

**Table S7: Binding sites and primer sequences for SH3PXD2A and CCR7 promoters.**
