# Supplementary figures and images for: Association of lncRNA SH3PXD2A-AS1 with preeclampsia and its function in invasion and migration of placental trophoblast cells
Source: Cell Death Dis. 2020 Jul 27;11(7):583. doi: 10.1038/s41419-020-02796-0 (PMC7385659; doi:10.1038/s41419-020-02796-0)

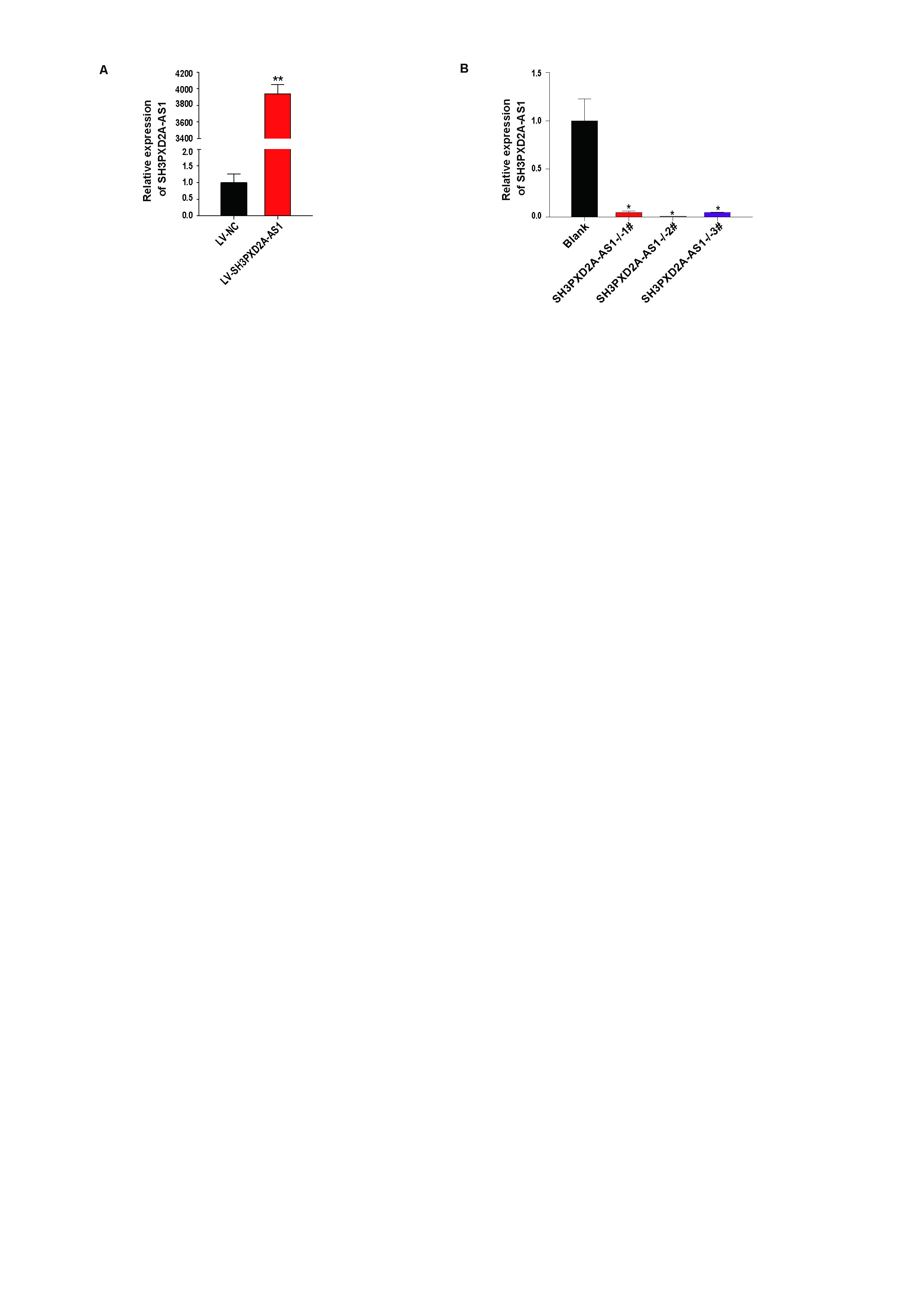

Supplement: Supplementary file 2 — Supplementary Figure S1 [file 41419_2020_2796_MOESM2_ESM.tif]

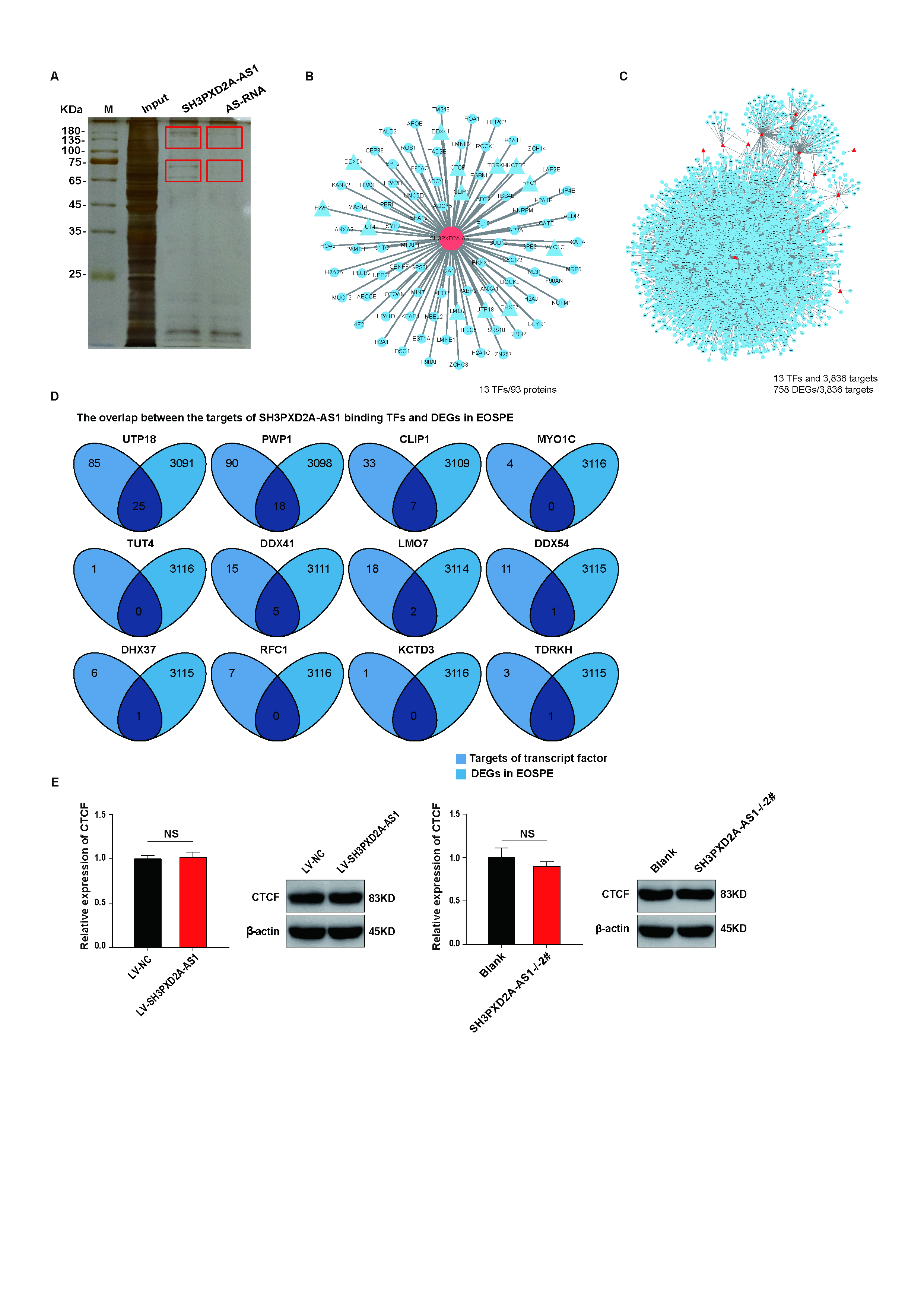

Supplement: Supplementary file 3 — Supplementary Figure S2 [file 41419_2020_2796_MOESM3_ESM.tif]

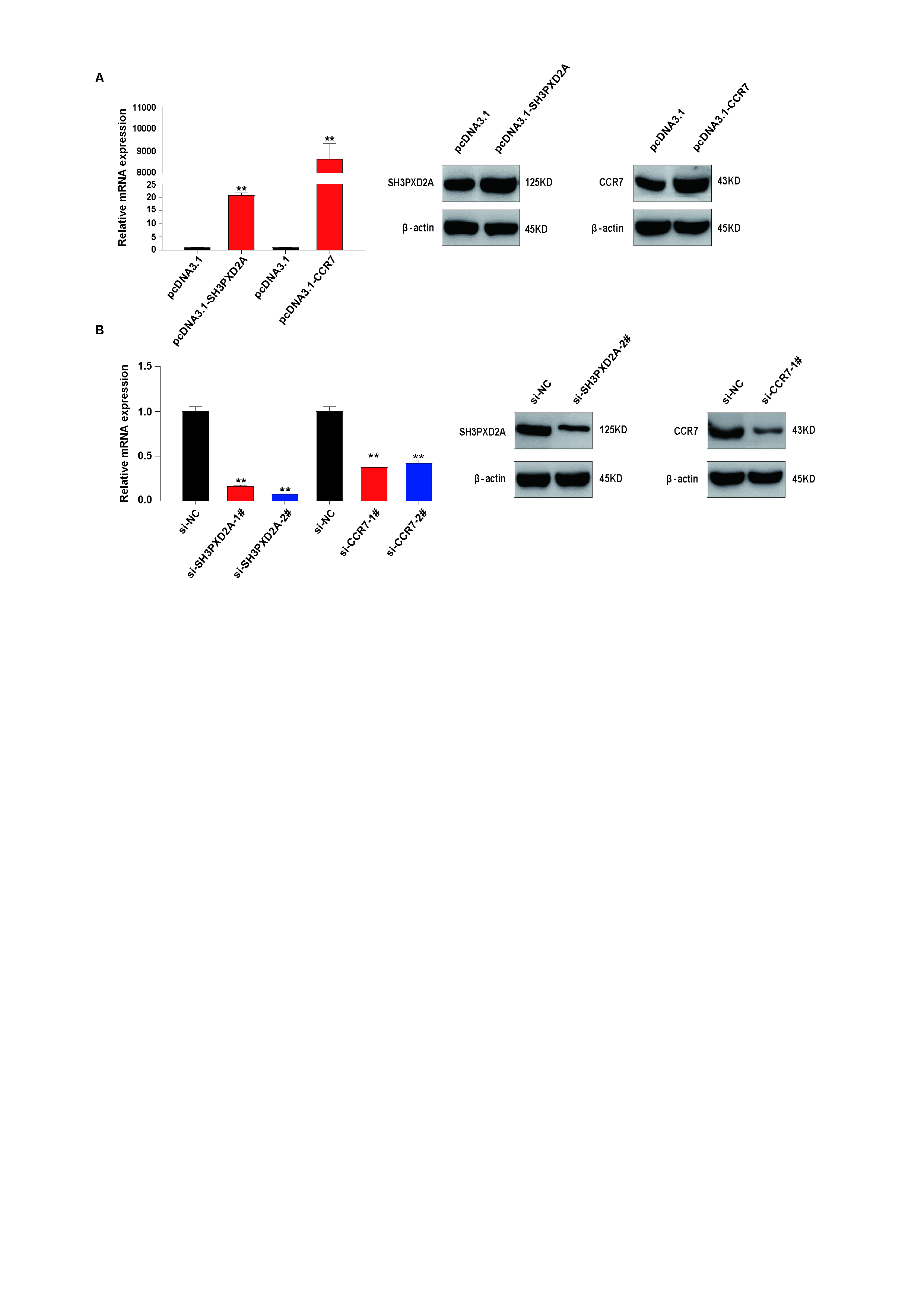

Supplement: Supplementary file 4 — Supplementary Figure S3 [file 41419_2020_2796_MOESM4_ESM.tif]

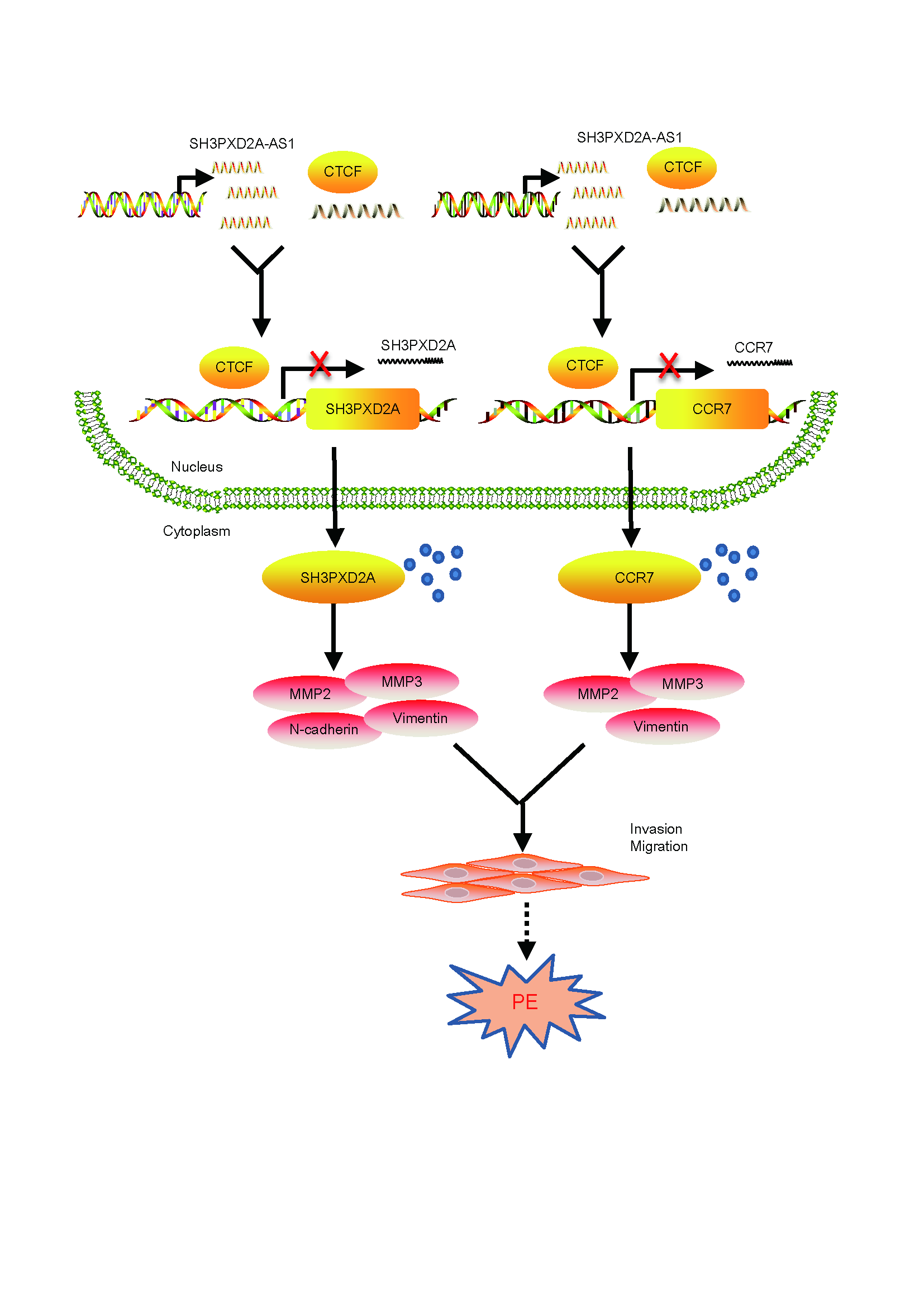

Supplement: Supplementary file 6 — Supplementary Figure S5 [file 41419_2020_2796_MOESM6_ESM.tif]
