# Supplementary material for: Association of lncRNA SH3PXD2A-AS1 with preeclampsia and its function in invasion and migration of placental trophoblast cells
Source: Cell Death Dis. 2020 Jul 27;11(7):583. doi: 10.1038/s41419-020-02796-0 (PMC7385659; doi:10.1038/s41419-020-02796-0)
Supplement: Supplementary file 12 — Supplementary Table S6 [file 41419_2020_2796_MOESM12_ESM.docx]

**Table S6**： Sequences of primers and siRNAs.

| Primers | Sense(5’-3’) | Antisense(5’-3’) |
| --- | --- | --- |
| SH-AS1 | GTTCGCTGTCCCTGTTCCT | CAATGGTGGCAGATTCTGGGA |
| ACTB | CTCCATCCTGGCCTCGCTGT | GCTGTCACCTTCACCGTTCC |
| sgRNA | CACCGTTTCAGCAAGTCGCGGTCG | AAACCGACCGCGACTTGCTGAAAC |
| SH-AS1-LA | CCATCGATTGACAGTCCAAATGGGTGCAG | CCGCTCGAGACTCTACCCGATGACTTTGCCT |
| SH-AS1-RA | ACGCGTCGACTGTTTCCCTGTTAGTTCCTGGC | CGCGGATCCCAGTAGACCTGCTGTCTTATCCC |
| Insert | AATCCCTCCTTCATTCTCAGTCCA | AGGCCACTTGTGTAGCGCCA |
| Double knock | TAAACTCCTGGGCTCAAGCGA | CCTGGGCGACATAGCAAGAC |
| SH3PXD2A | AAGAACGAGAGCGGCTGG | TCTTCCGGATCACCTCCACT |
| CCR7 | AAGCGATGCGATGCTCTCTC | TTGCGCTCAAAGTTGCGTG |
| SPAG4 | TCTCCAGTAGTCTCTGAGGAGC | CGGATGGAACAGACCTCCC |
| GPNMB | CTTCTGCTTACATGAGGGAGC | GGCTGGTGAGTCACTGGTC |
| DUSP1 | ACCACCACCGTGTTCAACTTC | TGGGAGAGGTCGTAATGGGG |
| RARRES1 | TCAGCATACCTGATAATCATGGACA | AGGTTTTTCTTACCCACTGCCT |
| BCL6 | ACACATCTCGGCTCAATTTGC | AGTGTCCACAACATGCTCCAT |
| KIT | CGTTCTGCTCCTACTGCTTCG | CCCACGCGGACTATTAAGTCT |
| FSTL3 | GTGCCTCCGGCAACATTGA | GCACGAATCTTTGCAGGGA |
| PROCR | GAGTGGTCACCTTCACCCTG | GCTTGTTTGGCTCCCTTTCG |
| CTCF | CAGTGGAGAATTGGTTCGGCA | CTGGCGTAATCGCACATGGA |
| GAPDH | GACCTGACCTGCCGTCTA | AGGAGTGGGTGTCGCTGT |
| U6 | CTCGCTTCGGCAGCACA | AACGCTTCACGAATTTGCGT |
| MMP2 | TACAGGATCATTGGCTACACACC | GGTCACATCGCTCCAGACT |
| MMP3 | CGGTTCCGCCTGTCTCAAG | CGCCAAAAGTGCCTGTCTT |
| Vimentin | GACGCCATCAACACCGAGTT | CTTTGTCGTTGGTTAGCTGGT |
| N-cadherin | AGCTCCATTCCGACTTAGACA | CAGCCTGAGCACGAAGAGTG |
| U1 | GGGAGATACCATGATCACGAAGGT | CCACAAATTATGCAGTCGAGTTTCCC |
| siRNA | Sense(5’-3’) | Antisense(5’-3’) |
| SH3PXD2A-1 | GGAGGUACAGCAAGUUCUUTT | AAGAACUUGCUGUACCUCCTT |
| SH3PXD2A-2 | GCCAAGGAUGACCUGCCAATT | UUGGCAGGUCAUCCUUGGCTT |
| CCR7-1 | GGACGUGCGGAACUUUAAATT | UUUAAAGUUCCGCACGUCCTT |
| CCR7-2 | GCAACUUUGAGCGCAACAATT | UUGUUGCGCUCAAAGUUGCTT |
