# Supplementary material for: Association of lncRNA SH3PXD2A-AS1 with preeclampsia and its function in invasion and migration of placental trophoblast cells
Source: Cell Death Dis. 2020 Jul 27;11(7):583. doi: 10.1038/s41419-020-02796-0 (PMC7385659; doi:10.1038/s41419-020-02796-0)
Supplement: Supplementary file 13 — Supplementary Table S7 [file 41419_2020_2796_MOESM13_ESM.docx]

**Table S7:** Binding sites and primer sequences of SH3PXD2A and CCR7 promoters.

| Gene | Binding sites |
| --- | --- |
| SH3PXD2A-1 | chr10:103855832-103856673 |
| SH3PXD2A-2 | chr10:103859416-103860013 |
| SH3PXD2A-3 | chr10:103861004-103861577 |
| CCR7-1 | chr17:40565493-40566957 |
| CCR7-2 | chr17:40581671-40582408 |
| CCR7-3 | chr17:40582478-40583039 |

| Promoter | Sense(5’-3’) | Antisense(5’-3’) |
| --- | --- | --- |
| SH3PXD2A -1 | ggcttggaaagtgggttttc | cgacagggaaaggagcataca |
| SH3PXD2A -2 | actctcaagggaccgtgtaaagaa | catactaacaggcccctcctgtt |
| SH3PXD2A -3 | agttttcaaaaacaaataaataaatgcctta | ttcaagttactattctttaaatatttattcagcatta |
| CCR7-1 | acaaacacttgcacataaatatttatagca | gctgaataacattccattgtatggatat |
| CCR7-2 | caaatataattcctaatactcaccagacaaca | agccagatgaagacatcccaa |
| CCR7-3 | caccacagaggttatttcagtcca | gcccagagcttttgtgcat |
| GAPDH | CATGGGTGTGAACCATGAGA | GTCTTCTGGGTGGCAGTGAT |
